# Characterization of a Mouse Model of Inducible Frailty: The Humanized IL-6 Mouse

**DOI:** 10.1093/geroni/igab046.2043

**Published:** 2021-12-17

**Authors:** Lolita Nidadavolu, Peter M Abadir, Jeremy Walston, Anne Le, Gayane Yenokyan, Liliana Florea, Corina Antonescu, D Brian Foster

**Affiliations:** 1 Johns Hopkins University, Baltimore, Maryland, United States; 2 Johns Hopkins University School of Medicine, Baltimore, Maryland, United States; 3 Johns Hopkins Bloomberg School of Public Health, Baltimore, Maryland, United States

## Abstract

The cytokine interleukin-6 (IL-6) has pleiotropic effects in aging and is elevated in frail older adults. We have developed a conditional mouse model to better characterize the role of IL-6 in promoting frailty and age-related mitochondrial dysregulation. The human IL-6 (hIL-6) knock-in mouse (TetO-hIL6) was developed utilizing CRISPR/Cas9 technology with transgene donor vector containing a tetracycline response element promoter driving expression of hIL-6 cDNA. Male TetO-hIL6 mice were treated with doxycycline-containing water for six weeks starting at 8 months old. RNAseq analysis of whole blood demonstrated significant upregulation of pro-inflammatory related markers at 6 weeks compared to baseline and upregulated cell proliferation and metabolism pathways. Physical testing of TetO-hIL6 mice before and after hIL-6 induction demonstrated decreased grip strength (p =0.003), decreased running capacity (p = 0.02), and 40% increase in falls off of the treadmill (p = 0.001). Induced mice also demonstrated decreased basal body temperature (p < 0.001). Given the significant dysregulation of metabolism-related genes in RNAseq analysis and changes in basal body temperature following hIL-6 induction, we next performed untargeted metabolomics on plasma from mice at baseline and 6 weeks post-induction to better evaluate metabolic changes associated with hIL-6 elevation. We found changes in key serum metabolites, including circulating adenosine triphosphate (56% reduction, p = 0.02), pyruvate (35% reduction, p = 0.0006), alpha-ketoglutarate (47% reduction, p = 0.04), and succinate (306% increase, p = 0.001). The TetO-hIL6 mouse model allows for induction of hIL-6 at various timepoints across the lifespan and demonstrates features of a frailty phenotype.

